# Uterine artery embolisation for symptomatic fibroids: the University of Malaya Medical Centre experience

**DOI:** 10.2349/biij.6.3.e27

**Published:** 2010-07-01

**Authors:** RN Subramaniam, A Vijayananthan, SZ Omar, O Nawawi, BJJ Abdullah

**Affiliations:** 1 Department of Obstetrics and Gynaecology, Faculty of Medicine, University of Malaya, Kuala Lumpur, Malaysia; 2 Department of Biomedical Imaging, Faculty of Medicine, University of Malaya, Kuala Lumpur, Malaysia

## Abstract

**Background:**

Transcatheter uterine artery embolisation (UAE) for the treatment of symptomatic fibroids has been performed in several centres in the United States, Western Europe and Asia with promising results. This study reports the authors' experience with UAE at the University Malaya Medical Centre.

**Method:**

Fifty women with symptomatic uterine fibroids who declined surgery were treated by transcatheter UAE. The uterine arteries were selectively catheterised and embolised with polyvinyl alcohol particles. Post-procedure analgesia was administered via patient-controlled analgesic pump. The patients were followed up at an interval of 6/12 clinically and with MRI.

**Results:**

Transcatheter UAE was performed on all 50 patients with no major complications. 49 patients had both uterine arteries embolised while 1 patient had only the right uterine artery embolised on account of hypoplasia of the left uterine artery due to previous myomectomy. The mean hospital stay was 3.5 days (range, 2 to 7). At a mean follow-up of 24/52, all patients reported improvements in their presenting symptoms. Objective improvement in terms of reduction of uterine and fibroid sizes was determined on MRI. One patient, who initially responded with a decrease in uterine and dominant fibroid size, became symptomatic (menorrhagia) after 6 months and subsequent endometrial sampling revealed cystic glandular hyperplasia for which total abdominal hysterectomy was performed. Two other patients had no change in symptoms and after hysterectomy, the pathology revealed concurrent adenomyosis. Another 2 patients with cervical fibroids were treated with hysterectomy as there was no gross reduction in the size of fibroid following UAE. Overall, 90% of the patients had dramatic improvement of anaemia and symptoms at 1 year follow-up.

**Conclusion:**

Out of the 50 patients, 17 patients had total disappearance of their fibroids and 28 patients had more than 50% reduction in the size of fibroids after 1 year. 5 patients ended up with total abdominal hysterectomy. These results suggest that UAE is an appealing alternative to hysterectomy or myomectomy for many women with symptomatic fibroids.

## INTRODUCTION

The most common tumour in women of the reproductive age group are uterine fibroids. They are symptomatic in about 50% of women and produce a variety of symptoms. These symptoms include menstrual disturbances, pelvic pain, pressure symptoms and compromised reproductive function [1]. The standard therapy for fibroids has been surgical removal by hysterectomy or myomectomy, if medical therapy fails.

In 1995, Ravina *et al*. introduced the use of transcatheter uterine artery embolisation. (UAE) as the primary treatment of uterine fibroids with encouraging results [2]. Since then, it has gained popularity as a minimally invasive, uterine-sparing procedure and currently more than 100,000 procedures have been performed worldwide [3,4]. UAE is not a new vascular intervention technique as it has been recognised as a superior first-line alternative to surgery for control of obstetric haemorrhage since the 1990s [5]. The utilisation of UAE for the primary treatment of fibroids is, however, a more recent phenomenon. This study reports preliminary experience with UAE in the local population as this has never been done before in this country.

## MATERIALS AND METHODS

The patients were recruited from the gynaecology clinic of the centre. All of them presented with symptomatic fibroids, having declined, or been deemed medically unfit for surgery. Their symptoms were found to be due to fibroids and were of sufficient severity to warrant surgical management.

The patients were selected, screened and investigated relevantly in the Department of Gynaecology and treated in collaboration with the Interventional Radiologists. All patients were treated according to the protocol approved by the institution’s ethics committee and with informed consent. The inclusion criteria included healthy pre-menopausal women between the ages of 38 and 55 years, not desirous of pregnancy, with large uteri (>12 weeks’ size) due to fibroids, and with at least one of the following symptoms: i) anaemia, ii) menorrhagia, iii) dysmenorrhoea, and iv) mass per abdomen with pressure symptoms. Underlying gynaecological malignancy was excluded by endometrial sampling.

The exclusion criteria included contraindications to Magnetic Resonance Imaging (MRI), severe allergy to iodinated contrast media, undiagnosed vaginal bleeding, subserosal pedunculated fibroids, patients on anticoagulation therapy or having clotting disorders, infection, immuno-compromised patients and patients who had not completed their family. A history of current and previous medical therapy for fibroids was recorded. An ultrasound of the pelvis was initially performed at the Gynaecology clinic to confirm the presence of uterine fibroids. An MRI using a 1.5 Tesla Siemens Magnetom Vision machine was subsequently carried out as a pre-requisite to the procedure. The MRI protocol used was a T1- and T2-weighted axial, T2-weighted sagittal and post-contrast T1-weighted axial and sagittal images. The patients who presented with menorrhagia were analysed based on the haemoglobin levels and duration of menorrhagia (use of sanitary napkins exceeding 12 per day).

Pre-procedural blood tests included full blood count, coagulation profile, as well as creatinine and electrolyte levels. All the selected cases were discussed with the interventional radiologist. The patients were also referred to the Acute Pain Service for evaluation by an anaesthetist and instructed on the use of patient-controlled analgesia (PCA).

The procedure was performed during the first 10 days of the patient’s menstrual cycle, after carefully excluding pregnancy. A urine pregnancy test was carried out in all sexually active patients in the reproductive age group. The patient was admitted a day before the procedure and was required to fast for 6 hours prior to the procedure. Prophylactic broad-spectrum intravenous antibiotic (Cefuroxime) was administered an hour before the procedure. Intra-procedural sedation and analgesia were administered by the attending radiologist. The standard protocol for analgesia was the administration of a bolus dose of 3 mg of morphine before the start of the procedure followed by 2 mg before embolisation of each side.

The right common femoral artery was punctured using a one-part needle and a 5F Robert’s uterine catheter was advanced into the left uterine artery. A diagnostic contrast run was done to evaluate the vascularity of the fibroid and to identify the ovarian arteries. The tip of the catheter was then advanced beyond the origin of the ovarian artery. Subsequently the left uterine artery was embolised with polyvinyl alcohol (PVA) particles (300 to 500 microns in size). The procedure was repeated on the right side. There were no microcatheters used in all our patients. A post-embolisation run was done prior to removal of the catheter.

All patients were observed as in-patients for at least 24 hours after the procedure. The PCA was set at morphine 1mg/bolus with a 5-minute lockout at a maximum of 10mg/h. The amount of morphine required, length of hospital stay and occurrence of any complication was noted. They were discharged with non-steroidal anti-inflammatory drugs (NSAIDs) or mefenamic acid of a week's duration. After discharge from the hospital, the patients were reviewed at 3, 6 and 12 months respectively. The severity of the presenting symptoms was reassessed. A follow-up MRI was performed at 6 and 12 months.

## RESULTS

Fifty women aged between 38 and 52 years (mean, 43) were referred for UAE for the treatment of symptomatic fibroids. Menorrhagia was present in 35 (70%) women, dysmenorrhoea in 12 (24%) women, pressure symptoms in 3 (6%) women, and anaemia in 32 (64%) women. The mean haemoglobin level was 9.9 mg/dL. The subjects had previously received medical treatment, namely hormonal therapy (progestogen) and NSAIDs. Six patients had undergone previous myomectomy. There were eight women with chronic medical disorders such as diabetes mellitus, hypertension, bronchial asthma, thalassaemia minor and idiopathic thrombocytopenic purpura (ITP).

Forty percent of the women had received previous treatment with blood transfusions, oral progestogens (NET), gonadotropin (zoladex) and previous myomectomy (six women). Ninety percent of the patients reported that menorrhagia had a heavy impact on their lifestyle in terms of interfererence with sleep, being housebound or having to be close to a washroom during menstruation.

UAE was successfully performed on all patients. 49 patients had both uterine arteries embolised; while 1 patient had only the right uterine artery embolised on account of hypoplasia of the left uterine artery.

The mean procedure time was 60 min (range, 30 to 90). There were no complications associated with the procedure itself (contrast media, groin haematomas). Nine patients developed fever and four developed urinary tract infection. Two of the patients developed blood-stained discharge for about a week after the procedure. Post-procedural pain was well-controlled in all women. The mean hospital stay was 3.5 days (range, 2 to 7). All patients were followed up at 3, 6 and 12 months. 90% of the patients reported improvement in the presenting symptoms. On follow-up at the clinic, they were questioned regarding their menstrual cycle, change in lifestyle and overall well-being. A standard quality-of-life score was not utilised. Objective improvement, in terms of reduction of uterine and fibroid sizes, was determined by MRI. There was more than 50% reduction in the size of the fibroid in 28 (56%) patients and total disappearance of the fibroid in 17 (34%) participants. Uterine and fibroid volumes were calculated based on MRI including maximum diameters in three planes (longitudinal [D1], anterior posterior [D2], and transverse [D3]). Measurements were only taken of the dominant or largest fibroid. Uterine and fibroid volumes were calculated using the formula V=0.5233xD1xD2xD3. The number and location of fibroids were also recorded. Majority of the women who participated in this study had single fibroid (39 women) and about 11 women had more than 5 fibroids. Pre-embolisation uterine volume was about 520 cm^3^.

Five patients had total abdominal hysterectomy following UAE. Two patients had concurrent adenomyosis, one had persistent menorrhagia with cystic glandular hyperplasia and two patients with cervical fibroid showed no reduction in the size with persistence of pressure symptoms. Two women, aged 47 and 52 years, experienced transient amenorrhoea at 12 and 24 weeks, respectively, after UAE, but did not have symptoms associated with menopause. The transient amenorrhoea in both these patients lasted for about 9 months and they had resumed their normal menstrual cycle at the one-year follow-up.

## DISCUSSION

Uterine leiomyomata is a major health problem that afflicts at least about 20-40% of women in the reproductive age group worldwide. The symptoms vary from abnormal bleeding to pain and pressure symptoms [3]. The true prevalence of fibroids is relatively unknown as they are only symptomatic in about 50% of women who develop them [6]. About 20-30% of hysterectomies are performed as a treatment for uterine fibroids. Another alternative treatment option is the use of gonadotrophin-releasing hormone agonists, either alone or in combination with conservative surgical treatments like myomectomy [7]. However, studies show that there is a significant rate (20-30%) of recurrence in fibroids after a myomectomy [8]. In fact, in this series, 6 women had previous myomectomy with recurrence of fibroids and symptoms.

Over the past decade, uterine artery embolisation (UAE) has been described as an alternative to invasive surgical procedures. Many studies have addressed this treatment option over the past 10 years. UAE is a percutaneous, image-guided procedure which is performed by a trained interventional radiologist. It involves the placement of a catheter into the uterine arteries via a common femoral artery approach and injection of embolic material (polyvinyl alcohol particles or gelfoam) into both uterine arteries until the flow becomes sluggish [2, 9-12]. The mechanism by which UAE acts is by reducing blood flow to the fibroid thus inducing irreversible ischaemia which leads to necrosis and shrinkage. The normal myometrium, however, recovers well from this ischaemic event [12].

The procedure generally takes about an hour. Immediately after the procedure, most patients experience cramping pain. This ischaemic pain generally lasts for 8-12 hours post-procedure [12]. Patients are usually kept overnight for pain management. In this centre, patients are instructed on the use of Patient Controlled Analgesia which is administered by an anaesthetist. The study also follows a protocol of 3 mg of morphine i/v before the start of the procedure and 2 mg before embolisation of each side. This protocol has been tolerated well by the patients in this series and they have been pain-free during the first 24 hours. They are discharged home with oral anaelgesics and do not report any pain-related complications on follow-up.

Another issue to consider is that of fertility. The effect of UAE on subsequent fertility and pregnancy has been understudied and therefore it is not recommended in women desirous of future pregnancies [13]. Transient or permanent amenorrhea, with symptoms of ovarian failure, have been reported in about 5% of patients after UAE. This has been attributed to ovarian embolisation via collaterals resulting in ovarian ischaemia [14]. Due to this concern, many studies were carried out to evaluate ovarian reserve by measuring the FSH levels before and after UAE. It was reassuring that there was no change detected in the ovarian reserve, but long-term studies have been recommended to further address this issue [15]. In this study, all patients who had not completed their family were excluded.

There are some limitations in this study that have to be addressed. Firstly, the follow-up period of 1 year is too short. Most of the patients did not return for a follow-up after the 1-year period and there is no data regarding their long-term clinical outcome. According to Hehenkamp *et al*. (2008), about 25% of patients in the EMMY trial had secondary hysterectomy after UAE at 24 months [16]. Currently, there are only 7 patients, out of the 50 in this series, who are still on regular follow-up and are doing well. Secondly, this study did not use a standard quality-of-life (QOL) questionnaire on follow-up. The patients were asked about their symptoms based on their menstrual cycle history. The authors recommend that a standard QOL score be done for further studies in the future.

## CONCLUSION

The mid-term results of UAE for the treatment of symptomatic fibroids in our hospital indicate this to be a safe and effective therapeutic option. A longer period of follow-up with a greater number of patients in this ongoing study will be needed to confirm that UAE is a viable option, acceptable to both patients and clinicians. This study illustrates that large numbers of women with symptomatic fibroid disease are averse to surgery despite their burden of suffering, and are actively seeking alternatives to hysterectomy. UAE is a nonsurgical option for management of fibroid related symptoms that has shown excellent technical and clinical success. Although there have been many similar studies done globally over the past 10 years, there has not been any published South-East Asian series and the authors feel that this preliminary experience would be helpful to clinicians in this region.

**Table 1:** The outcome at 6 months clinical follow-up

**Symptoms at 6 Month Follow-up**	**No. of Patients**
Scanty Menses	19
Normal Menses	24
Transient Amenorrhea	2
Persistent Menorrhagia	5

**Figure 1:**
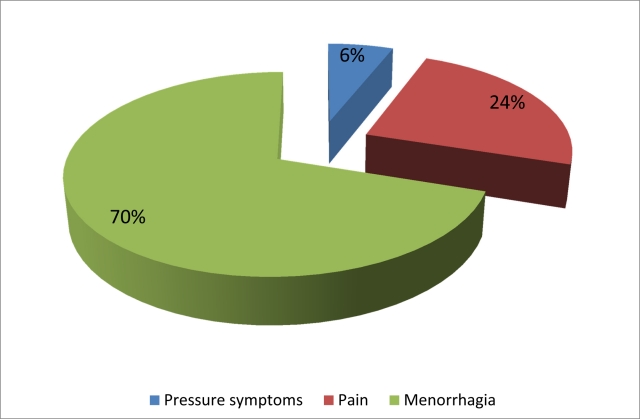
Prevalence of symptoms: 70% of the patients presented with menorrhagia, 24% presented with pain and 6% presented with pressure symptoms. Concurrently about 64% of the patients were found to be anaemic.

**Figure 2:**
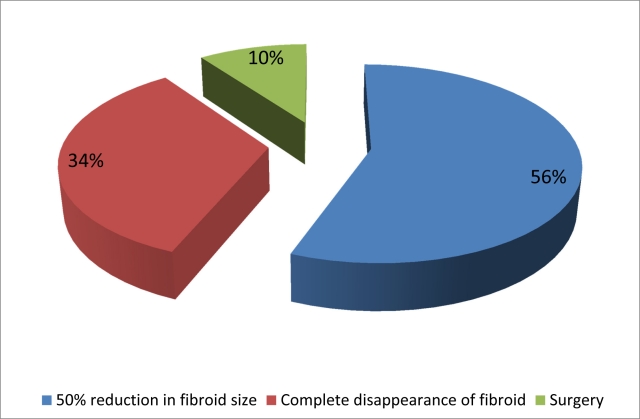
This figure shows the outcome of the 50 patients at 1-year follow up. 56% of patients had 50% reduction of volume as measured on MRI. 34% of the patients had complete absence of the fibroid and 10% had hysterectomy done.

**Figure 3:**
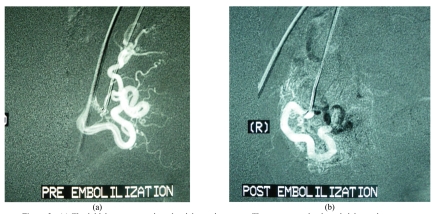
(a) The initial contrast run into the right uterine artery. The tortuous and enlarged right uterine artery and the rich blood supply to the fibroid can be seen; (b) the post-embolisation contrast run which shows staining of the fibroid and no opacification of the arteries seen in the previous run.

**Figure 4:**
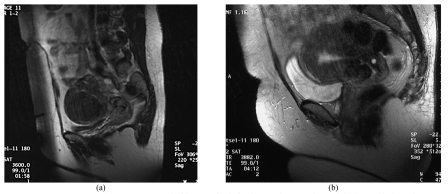
A 50-year-old woman presented with menorrhagia for the past three months. An initial MRI (a) showed the presence of an anterior intramural fibroid. She underwent a UAE and a follow-up MRI at 1 year (b) showed complete absence of the fibroid.

**Figure 5:**
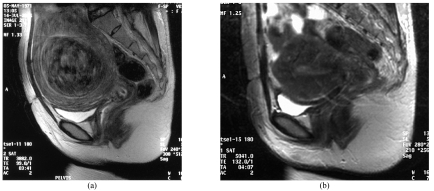
A 45-year-old woman presented with a two-month history of menorrhagia which required repeated blood transfusion. She was advised to have surgery but declined. Her initial MRI (a) showed the presence of a large submucosal fibroid. Due to her clinical status, a decision was taken to do a UAE. Her follow-up MRI at 1 year (b) shows complete absence of the fibroid.
